# Experiments on Temporary or Definitive Survivals Following Severe Hemorrhages

**Published:** 1919-01

**Authors:** 


***Experiments on Temporary or Definitive Survivals Following Severe Hemorrhages.*** By C. Richet and Fr. Saint-Girons. *Comptes Rendus de l'Académie des Sciences.* October 21, 1918.

Under the heading of temporary survivals, the authors consider such survivals as do not last over a few hours. For instance, dogs suffering from hemorrhage, with considerable respiratory distress and a very faint heart, seem really to revive after an abundant injection of some saline solutions. They are able to stand, and even walk and run about, but those symptoms do not last, and after a few hours these dogs finally die.

In cases of hemorrhage, the blood is rapidly diluted by the interstitial fluids of the organism. Consequently the amount of fluid blood escaped from an organism does not measure exactly the real loss of blood; it is the loss in red blood cells that must be taken into consideration.

A dog dies of hemorrhage when he has lost 75 o/o of his red blood cell count (average result of 12 experiments); but long before that lethal limit is reached, the general distress is so great that the dog dies if no intervention occurs.

In order to determine the proportion of red blood cells lost, it is generally admitted that the weight of blood is 1/13 of the weight of the whole body. It may be so in most cases, but of course there are marked differences according to the individuals.

At any rate, the authors admit that such therapeutic means only can be considered as *truly efficient* against hemorrhage, as are followed by definitive survival after the loss of 70 per cent. of the total count of red blood cells.

Two series of experiments were undertaken. In one of these the dogs were given, after hemorrhage, injections with different solutions (horse serum, saline solution, whey).

In the other, the dogs suffering from hemorrhage were transfused with either citrated serum or plasma, or directly with whole blood from artery to vein.

(In order to avoid the toxic effects of sodium citrate in the organism, a concomitant injection of calcium hydrochloride was given, according to Sabattini's method. It must be noted that, in the cases of direct transfusion from artery to vein, recovery was most extraordinarily rapid and complete).

*Results.* — Out of 19 dogs which had kept less than 42 per cent. of their blood, and which were given injections of saline solutions, only 2 survived.

Among 17 which had kept less than 40 per cent. of their blood, and which were transfused, no death occurred.

Injections of saline solutions cannot be considered hereafter as an efficient treatment. The transfusion of blood or plasma is the only effective means of treatment.

The saline injections might perhaps be resorted to, in order to obtain a temporary survival and allow of some subsequent transfusion. But the good effects of transfusion seem to be impaired by a former saline injection. Out of 8 dogs, which were given both treatments, one only survived.

